# Reimbursement of Apps for Mental Health: Findings From Interviews

**DOI:** 10.2196/14724

**Published:** 2019-08-06

**Authors:** Adam C Powell, Matthias B Bowman, Henry T Harbin

**Affiliations:** 1 Payer+Provider Syndicate Boston, MA United States; 2 The Bowman Family Foundation Hockessin, DE United States

**Keywords:** mental health, psychiatry, compensation, mobile health, clinical coding, administrative claims, healthcare

## Abstract

**Background:**

Although apps and other digital and mobile health tools are helping improve the mental health of Americans, they are currently being reimbursed through a varied range of means, and most are not being reimbursed by payers at all.

**Objective:**

The aim of this study was to shed light on the state of app reimbursement. We documented ways in which apps can be reimbursed and surveyed stakeholders to understand current reimbursement practices.

**Methods:**

Individuals from over a dozen stakeholder organizations in the domains of digital behavioral and mental health, care delivery, and managed care were interviewed. A review of Current Procedural Terminology (CPT) and Healthcare Common Procedure Coding System (HCSPCS) codes was conducted to determine potential means for reimbursement.

**Results:**

Interviews and the review of codes revealed that potential channels for app reimbursement include direct payments by employers, providers, patients, and insurers. Insurers are additionally paying for apps using channels originally designed for devices, drugs, and laboratory tests, as well as via value-based payments and CPT and HCSPCS codes. In many cases, it is only possible to meet the requirements of a CPT or HCSPCS code if an app is used in conjunction with human time and services.

**Conclusions:**

Currently, many apps face significant barriers to reimbursement. CPT codes are not a viable means of providing compensation for the use of all apps, particularly those involving little physician work. In some cases, apps have sought clearance from the US Food and Drug Administration for prescription use as digital therapeutics, a reimbursement mechanism with as yet unproven sustainability. There is a need for simpler, more robust reimbursement mechanisms to cover stand-alone app-based treatments.

## Introduction

### Diversity of Apps

Numerous patient-facing and provider-facing smartphone apps are available to potentially improve the mental health of Americans. Apps are being used for screening, diagnosis, treatment, ongoing monitoring, decision making, and administrative purposes. As a result of the broad variety of apps in use, there are diverse means of reimbursement being used to compensate for app utilization.

Some mental health apps are being paid for by employers, insurers (public and private), health care providers, and patients. In general, insurers and employers are paying for apps in 2 ways: paying for them directly and paying for them indirectly by paying for services that are facilitated by app-based interventions. When insurers pay for apps directly, they are using multiple means to do so: reimbursing them through paying Current Procedural Terminology (CPT) or Healthcare Common Procedure Coding System (HCSPCS) codes, through paying for them as if they were pharmaceuticals or medical devices, and by making direct payments for them, which are not tied to any codes or defined payment mechanisms. To summarize the present state of app reimbursement, this paper provides an overview of how apps are being reimbursed today, documents potential pathways to reimbursement, and reviews the limitations of these pathways. Apps, like all medical products, warrant reimbursement only if they are effective. There are a number of methods by which app effectiveness can be evaluated. We do not address those methods here.

### Overview of How Apps Can Be Reimbursed Today

**Figure 1:**
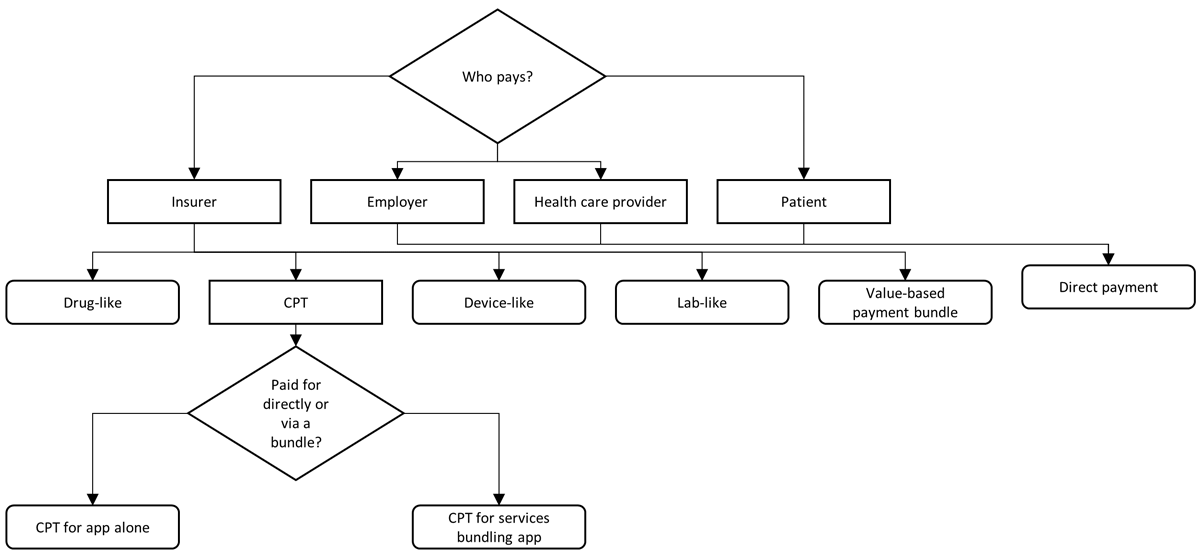
Channels for app reimbursement. CPT: Current Procedural Terminology.

App reimbursement is occurring through multiple channels, and some major categories of apps are not receiving reimbursement from payers at all. As is shown in Figure 1, there are at least 6 different pathways to reimbursement that exist. The diversity in potential reimbursement mechanisms is an outgrowth of both the wide set of actors paying for apps (insurers, employers, health care providers, and patients) and the variation in functionality of the apps themselves. Adding to the confusion, interviews with industry stakeholders revealed that in some situations, a single app is being reimbursed through multiple means, depending on the context. Thus, the means for reimbursement is neither universal across apps nor even always within a given app. A common means of funding apps is direct payment. When direct payments are made, employers, insurers, or health care providers can compensate the app vendor by paying a one-time fee for a general license to the app, paying a subscription for a general license to the app, paying a one-time fee per user, paying a per user-per month fee, paying a per employee/member per month fee, or paying a fee tied to the level of app utilization. Similarly, patients may directly buy access to apps for themselves on a one-time basis, via subscription or on the basis of utilization (eg, in-app purchases). When apps are not purchased directly, insurers may pay for them as if they were prescription drugs or devices and as if they were laboratory tests, or they may pay for them via payments made for CPT codes. Finally, money paid through value-based payment arrangements can be used by health care providers to purchase apps. Although this mode of payment is rather indirect, it does enable health care providers to purchase apps if they believe that doing so will decrease costs or enhance the value of care that they deliver.

**Figure 2:**
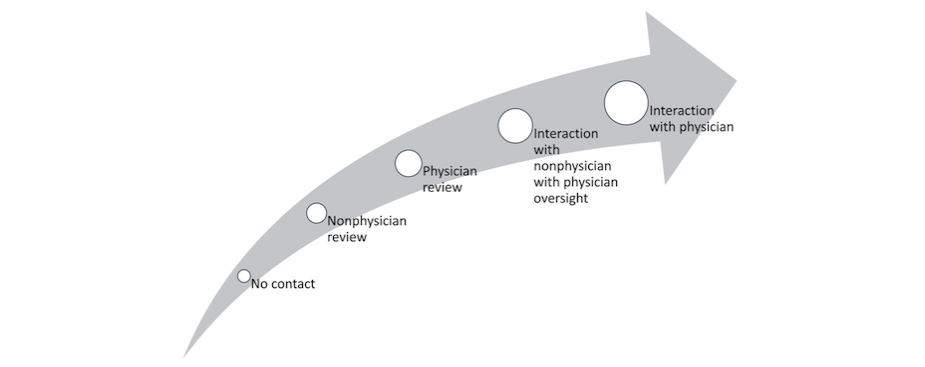
Spectrum of health care provider and technician involvement in app use.

When CPT codes are used to facilitate payment for apps, there are 2 ways in which they may be implemented. First, some CPT codes are directly applicable to an app-delivered health care service. For instance, app-based screening can fulfill the requirements of a screening CPT code. Second, some CPT codes are applicable to broad services that can be facilitated by apps. For example, an app-based platform might facilitate collaborative care and enable a health care provider to bill for the CPT code associated with collaborative care. Some of the revenue from the CPT code could be used to cover the app’s costs, whereas the remainder would likely need to be spent on associated services (eg, staffing) necessary to perform the services associated with the billed CPT code. One of the factors that drive the need for multiple reimbursement methods to be used to cover apps is that apps vary in their level of physician and nonphysician involvement. As is shown in Figure 2, there is a spectrum of levels of physician and nonphysician (eg, nurse, psychologist, technician) involvement in the use of apps. Some apps involve absolutely no human involvement, for instance, self-help tools that a family physician might wish to recommend to a patient expressing a minor health issue. From there, progressively greater levels of human involvement can occur, ranging from the periodic review of data captured in an app by a nonphysician (eg, a brief screening) to live interaction with a physician (eg, telemental health). Stand-alone interventions lend themselves to being reimbursed as if they were devices, drugs, or laboratory tests, as these analogues were all designed to be services that are reimbursable without physician, nurse, or technician contact.

### Screening and Repeat Measures for Standardized, Quantitative Clinical Outcomes

A literature review found that virtually all randomized controlled trials have shown that the frequent, timely provision of standardized patient-reported symptoms during psychotherapy encounters is associated with improved outcomes [1]. Apps are a natural tool for screening and repeat measures, as they enable patients to report their state to their health care providers from the comfort of their home. When billing for apps performing screening and repeat measures for standardized, quantitative clinical outcomes, the appropriate CPT code may vary in accordance with the instrument being administered. The American Academy of Pediatrics has produced a table (reproduced below as Table 1), which indicates the correspondence between instruments and CPT codes [2].

**Table 1:** Current Procedural Terminology codes appropriate for assorted instruments.

Instrument	Codes			
	96110	96127	96160	96161
Acute Concussion Evaluation (ACE)	—^a^	—	X	—
Ages and Stages Questionnaire—Third Edition	X	—	—	—
Ages and Stages Questionnaire—Social Emotional	—	X	—	—
Australian Scale for Asperger Syndrome (ASAS)	—	X	—	—
Beck Youth Inventory— Second Edition BYI-II	—	X	—	—
Behavior Assessment Scale for Children—2nd Edition	—	X	—	—
Behavior Rating Inventory of Executive Function	—	X	—	—
Conners Rating Scale	—	X	—	—
CRAFFT Screening Interview	—	—	X	—
Edinburgh Postnatal Depression Scale^b^ (EPDS)	—	—	—	X
Edinburgh Postnatal Depression Scale (EPDS)	—	X	—	—
Kutcher Adolescent Depression Scale (KADS)	—	X	—	—
Modified Checklist for Autism in Toddlers (MCHAT)	X	—	—	—
Patient Health Questionnaire (PHQ-2 or PHQ-9)	—	X	—	—
Parents’ Evaluation of Developmental Status (PEDS)	X	—	—	—
Pediatric Symptom Checklist (PSC)	—	X	—	—
Screen for Child Anxiety Related Disorders (SCARED)	—	X	—	—
Vanderbilt rating scales	—	X	—	—

^a^Not applicable.

^b^When billed under the infant and not the mother.

The most common CPT code deemed appropriate is 96127, which is defined as “Brief emotional/behavioral assessment, (eg, depression inventory, attention-deficit/hyperactivity disorder [ADHD] scale), with scoring and documentation, per standardized instrument.” In the case of pediatrics, a different code that relates to developmental screening may be more appropriate: 96110, “Developmental screening (eg, developmental milestone survey, speech and language delay screen), with scoring and documentation, per standardized instrument.” Finally, if a health risk assessment is used to assess for mental health issues, either 96160, “Administration of patient-focused health risk assessment instrument (eg, health hazard appraisal), with scoring and documentation, per standardized instrument,” or 96161, “Administration of caregiver-focused health risk assessment instrument (eg, depression inventory) for the benefit of the patient, with scoring and documentation, per standardized instrument,” should be billed, depending on whether the focus of the assessment is the patient or the caregiver [3].

### Collaborative Care Codes

Information technology has the potential to play a key role in supporting collaboration, information exchange, and planning. Codes to support the collaborative care model (CoCM) program were introduced by the Centers for Medicare and Medicaid Services in 2018. Although these codes cannot be billed for the use of a freestanding app, as collaborative care inherently involves human effort, apps can play a role in facilitating collaborative care and documenting that the necessary resources have been provided to fulfill the requirements of the codes being billed. The collaborative care codes provide funding for both less intensive and more intensive versions of collaborative care. The code 99484 was created to fund Behavioral Health Integration models of care other than CoCM, and the codes 99492, 99493, and 99494 were created to cover the activities of true CoCM programs. Eligibility for 99484 differs from the CoCM codes, making it suitable for environments with fewer staff resources [4]. Although 99484 only requires at least 20 min of clinical staff time, overseen by a physician or qualified health professional, the CoCM codes require a psychiatric consultant and behavioral health care manager to be a part of the care team, which provides 70 min of services in the first month and 60 min of services in subsequent months.

## Methods

Individuals from over a dozen stakeholders in digital behavioral and mental health domain were interviewed, representing organizations offering solutions, including a digital device (app as a medical device), an app intended to take the place of a drug, app-facilitated telemedicine, app-facilitated care coordination and case management, app- and device-facilitated sobriety testing, a peer therapy app, a patient remote monitoring service, and a suite of app-based care tools. Other stakeholders interviewed included individuals associated with a commercial payer, a Medicaid payer, an employee assistance program, a health care provider organization, and a medical professional society. In total, 22 interviews were conducted, of which 10 were with for-profits offering digital tools, 4 were with nonprofits related to mental health, 3 were with health care provider organizations, 3 were with academics, and 2 were with government entities. During each of the interviews, the stakeholders were asked about the pathways their organizations had used to achieve reimbursement, as well as the pathways through which they had seen other organizations achieve reimbursement. Payer and provider stakeholders were asked about how app-based care was financed by their organizations.

## Results

The list of codes in Table 2 summarizes the codes (1) that were found to be in active use or were attempted to be placed in active use during the interview process, or (2) that could theoretically be put into use in the judgment of the authors (these are noted as “not observed”). It contains HCSPCS codes, as defined by the Centers for Medicare and Medicaid Services; CPT codes are a subset of the HCSPCS codes. The types of providers (eg, psychiatrists, psychologists, social workers, and technicians) who may bill the codes vary from code to code. For instance, 96138 may be billed for work performed by a technician, whereas 96116 may only be billed for work performed by a “physician or other qualified health care professional.” To keep the list at a manageable length, the list of codes only contains those for initial encounters or for the lowest duration of a service. In some cases, there are related codes for subsequent encounters or longer durations of service. These codes were identified by reviewing the 2019 edition of the American Medical Association’s *CPT Professional* book, the definitive source on CPT codes [5].

It should be noted that some organizations reported using different codes with different payers and different codes in different clinical settings. For instance, a provider of a digital behavioral health integration and remote patient monitoring solution used the code 99489 in settings lacking a behavioral health specialist, leading to lower reimbursements, and 99492 in settings with a behavioral health specialist, leading to higher reimbursements. As was the case with the digital behavioral health integration tool, in many instances, some degree of health care provider involvement is necessary to bill a code. Thus, there were many cases in which digital tools played a key role in a solution, but they could not be reimbursed if used on an entirely stand-alone basis by a patient. Finally, many organizations are not using codes at all; instead, they are relying on other mechanisms, such as (1) direct billing via a “one-off” contract with an employer or insurer or (2) patient self-pay. These 2 approaches, used because of the absence of a code intended for apps per se, are cumbersome and difficult to implement on a large scale. They are an indication of the barrier to widespread adoption of apps that exists today.

**Table 2:** List of potential Healthcare Common Procedure Coding System (Current Procedural Terminology) codes that may potentially be used to reimburse for app-related services.

Code	Definition [3]	Context in which use of code was observed
90832	Psychotherapy, 30 min with patient (using 95 or GT modifier to indicate telemental health)	Teletherapy service
90885	Psychiatric evaluation of hospital records, other psychiatric reports, psychometric and/or projective tests, and other accumulated data for medical diagnostic purposes	Not observed
90887	Interpretation or explanation of results of psychiatric, other medical examinations and procedures, or other accumulated data to family or other responsible persons, or advising them how to assist patient	Not observed
90889	Preparation of report of patient’s psychiatric status, history, treatment, or progress (other than for legal or consultative purposes) for other individuals, agencies, or insurance carriers	Not observed
96105	Assessment of aphasia (includes assessment of expressive and receptive speech and language function, language comprehension, speech production ability, reading, spelling, and writing, eg, by Boston Diagnostic Aphasia Examination), with interpretation and report per hour	Cognitive and psychological screening app
96110	Developmental screening (eg, developmental milestone survey, speech, and language delay screen), with scoring and documentation, per standardized instrument	Not observed
96116	Neurobehavioral status exam (clinical assessment of thinking, reasoning and judgment, eg, acquired knowledge, attention, language, memory, planning and problem solving, and visual spatial abilities) by physician or other qualified health care professional, both face-to-face time with the patient and time interpreting test results and preparing the report; first hour	Not observed
96127	Brief emotional/behavioral assessment (eg, depression inventory, attention-deficit/hyperactivity disorder scale), with scoring and documentation, per standardized instrument	Screening component (eg, Patient Health Questionnaire-9, General Anxiety Disorder-7) within various self-service, patient-facing apps
96130	Psychological testing evaluation services by a physician or other qualified health care professional, including integration of patient data, interpretation of standardized test results and clinical data, clinical decision making, treatment planning and report, and interactive feedback to the patient, family member(s) or caregiver(s), when performed; first hour	Tech-enabled care management and case management service
96138	Psychological or neuropsychological test administration and scoring by a technician, 2 or more tests, any method; first 30 min	Tech-enabled care management and case management service
96146	Psychological or neuropsychological test administration, with single automated, standardized instrument via electronic platform, with automated result only	Tech-enabled care management and case management service
96160	Administration of a patient-focused health risk assessment instrument (eg, health hazard appraisal), with scoring and documentation, per standardized instrument	Not observed
96161	Administration of a caregiver-focused health risk assessment instrument (eg, depression inventory) for the benefit of the patient, with scoring and documentation, per standardized instrument	Medicaid depression initiative
99091	Collection and interpretation of physiologic data (eg, electrocardiogram, blood pressure, and glucose monitoring) digitally stored and/or transmitted by the patient and/or caregiver to the physician or other qualified health care professional, qualified by education, training, and licensure/regulation (when applicable), requiring a minimum of 30 min of time, every 30 days	Physiologically based sobriety monitoring program (exploring but not using code)
99358	Prolonged evaluation and management service before and/or after direct patient care; first hour	Not observed
99367	Medical team conference with interdisciplinary team of health care professionals, patient, and/or family not present, 30 min or more; participation by physician	Not observed
99401	Preventive medicine counseling and/or risk factor reduction intervention(s) provided to an individual (separate procedure); approximately 15 min	Health coaching
99406	Smoking and tobacco use cessation counseling visit; intermediate, greater than 3 min, up to 10 min	Between-visit patient remote monitoring and behavioral health integration app
99408	Alcohol and/or substance (other than tobacco) abuse structured screening (eg, Alcohol Use Disorders Identification Test, Drug Abuse Screening Test) and brief intervention services; 15 to 30 min	Not observed
99429	Unlisted preventive medicine service	Not observed
99446	Interprofessional telephone/internet/electronic health record assessment and management service provided by a consultative physician, including a verbal and written report to the patient’s treating/requesting physician or other qualified health care professional; 5-10 min of medical consultative discussion and review	Not observed
99453	Remote monitoring of physiologic parameter(s), for example, weight, blood pressure, pulse oximetry, and respiratory flow rate, initial; setup and patient education on use of equipment	Not observed
99457	Remote physiologic monitoring treatment management services, 20 min or more of clinical staff/physician/other qualified health care professional time in a calendar month, requiring interactive communication with the patient/caregiver during the month	Not observed
99483	Assessment of and care planning for a patient with cognitive impairment, requiring an independent historian, in the office or other outpatient, home or domiciliary or rest home, with all of the following required elements: cognition-focused evaluation, including a pertinent history and examination; medical decision making of moderate or high complexity; functional assessment (eg, basic and instrumental activities of daily living), including decision-making capacity; use of standardized instruments for staging of dementia (eg, functional assessment staging test, clinical dementia rating); medication reconciliation and review for high-risk medications; evaluation for neuropsychiatric and behavioral symptoms, including depression, including use of standardized screening instrument(s); evaluation of safety (eg, home), including motor vehicle operation; identification of caregiver(s), caregiver knowledge, caregiver needs, social supports, and the willingness of caregiver to take on caregiving tasks; development, updating or revision, or review of an Advance Care Plan; creation of a written care plan, including initial plans to address any neuropsychiatric symptoms, neurocognitive symptoms, functional limitations, and referral to community resources as needed (eg, rehabilitation services, adult day programs, and support groups) shared with the patient and/or caregiver with initial education and support. Typically, 50 min are spent face to face with the patient and/or family or caregiver	Not observed
99484	Care management services for behavioral health conditions, at least 20 min of clinical staff time, directed by a physician or other qualified health care professional, per calendar month, with the following required elements: initial assessment or follow-up monitoring, including the use of applicable validated rating scales; behavioral health care planning in relation to behavioral/psychiatric health problems, including revision for patients who are not progressing or whose status changes; facilitating and coordinating treatment, such as psychotherapy, pharmacotherapy, counseling and/or psychiatric consultation; continuity of care with a designated member of the care team	Between-visit patient remote monitoring and behavioral health integration app facilitating collaborative care; used in contexts where there is no behavioral health specialist
99487	Complex chronic care management services, with the following required elements: multiple (2 or more) chronic conditions expected to last at least 12 months or until the death of the patient, chronic conditions place the patient at significant risk of death, acute exacerbation/decompensation, or functional decline, establishment or substantial revision of a comprehensive care plan, moderate- or high-complexity medical decision making; 60 min of clinical staff time directed by a physician or other qualified health care professional, per calendar month	Tech-enabled care management and case management service
99490	Chronic care management services, at least 20 min of clinical staff time directed by a physician or other qualified health care professional, per calendar month, with the following required elements: multiple (2 or more) chronic conditions expected to last at least 12 months or until the death of the patient; chronic conditions place the patient at significant risk of death, acute exacerbation/decompensation, or functional decline; comprehensive care plan established, implemented, revised, or monitored	Tech-enabled chronic care management service
99492	Initial psychiatric collaborative care management, first 70 min in the first calendar month of behavioral health care manager activities, in consultation with a psychiatric consultant, and directed by the treating physician or other qualified health care professional, with the following required elements: outreach to and engagement in treatment of a patient, directed by the treating physician or other qualified health care professional; initial assessment of the patient, including administration of validated rating scales, with the development of an individualized treatment plan; review by the psychiatric consultant, with modifications of the plan if recommended; entering patient in a registry and tracking patient follow-up and progress using the registry, with appropriate documentation, and participation in weekly caseload consultation with the psychiatric consultant; provision of brief interventions, using evidence-based techniques, such as behavioral activation, motivational interviewing, and other focused-treatment strategies	Between-visit patient remote monitoring and behavioral health integration app facilitating collaborative care; used in contexts where there is a behavioral health specialist
99494	Initial or subsequent psychiatric collaborative care management, every additional 30 min in a calendar month of behavioral health care manager activities, in consultation with a psychiatric consultant, and directed by the treating physician or other qualified health care professional (listed separately, in addition to code for primary procedure)	Not observed
99495	Transitional Care Management Services, with the following required elements: communication (direct contact, telephone, and electronic) with the patient and/or caregiver within 2 business days of discharge. Medical decision making of at least moderate complexity during the service period. Face-to-face visit, within 14 calendar days of discharge	Not observed
99499	Unlisted evaluation and management service	Not observed
A9999	Miscellaneous Durable Medical Equipment (DME) supply or accessory, not otherwise specified	Treatment for substance use disorder (unclear if in active use)
E1399	Durable medical equipment, miscellaneous	Treatment for substance use disorder
G0444	Annual depression screening, 15 min	Medicaid depression initiative
G8431	Screening for depression is documented as being positive, and a follow-up plan is documented	Medicaid depression initiative
G8510	Screening for depression is documented as negative, a follow-up plan is not required	Medicaid depression initiative
H0047	Alcohol and/or other drug abuse services, not otherwise specified	Treatment for substance use disorder (vendor had difficulty obtaining payment from payers using this code)
T1505	Electronic medication compliance management device, includes all components and accessories, not otherwise classified	Treatment for substance use disorder

## Discussion

### Potential List of Codes That Could Be Used for Reimbursement

Given that a number of apps and digital tools for behavioral health are components of broader solutions involving clinicians or technicians and are only able to achieve reimbursement because of some level of clinician or technician involvement, every code for behavioral health could potentially be used to help cover the costs of a digital intervention. Furthermore, 96127, brief emotional/behavioral assessment, appears to be the main code used for app-based patient assessments in which there is no active clinician involvement while the assessment is being administered.

### Limitations of the Existing Reimbursement Pathways

In theory, almost any intervention could be shoehorned into one of the pathways depicted in Figure 1, especially if paired with physician, nonphysician health care provider, or technician time. Furthermore, shoehorning interventions into the “Direct Payment” pathway is problematic, as it requires negotiations to occur 1 payer, provider, or employer at a time. As such, it is not an efficient way for the adoption of an intervention to rapidly occur. Meanwhile, CPT and HCSPCS codes, offer a smoother pathway to reimbursement, but they do so only when payers are willing to honor them and provide sufficient reimbursements.

### Apps as Procedures

CPT codes are reimbursed by payers in accordance with the total number of Relative Value Units (RVUs) that they have been assigned. RVUs are a standardized unit, used across procedures to determine the amount of effort and expenditure involved in delivering a given procedure. There are 3 components to the total RVUs assigned to a CPT code: the work RVUs, malpractice RVUs, and practice-expense RVUs. Work RVUs capture the effort of the clinician before, during, and after the procedure. Practice-expense RVUs capture the supplies used to perform the procedure, as well as the associated costs of staff and the facility in which the procedure is performed. Malpractice RVUs are based on the degree of liability that the clinician incurs by performing the procedure, and these are calculated by using malpractice premium data [6]. To determine the total RVUs for a procedure, the 3 components are adjusted by a Geographic Practice Cost Index to account for geographic cost variation, and these are then combined. As the majority of the total RVU value ascribed to most CPT codes comes from the work RVU component, apps that do not involve physician work inherently lead to lower total RVUs. CPT codes for services performed without physician intervention, such as 96127, brief emotional/behavioral assessment, are primarily paying for the brief physician time involved in assimilating the information from assessment into the overall care plan. The total RVUs assigned to 96127, brief emotional/behavioral assessment, when performed in a nonfacility setting are low, as the procedure is worth 0.00 work RVUs, 0.01 malpractice RVUs, and 0.14 practice-expense RVUs. Given that Medicare paid in 2019 is US $36.04 per RVU, a brief emotional/behavioral assessment worth 0.15 RVUs would yield a payment of US $5.41. Although this payment may be adequate if a provider can efficiently assign the assessment, document its completion, assimilate the findings, and bill the appropriate CPT code, providers without efficient systems in place may have difficulty billing this code profitably.

The focus of using CPT codes to tie reimbursement largely to physician effort has resulted in there being a dearth of codes for services performed without physician intervention. Although there are a handful of codes for services provided without physician effort, such as those for transcranial magnetic stimulation or the administration of a health risk assessment, a vast majority of CPT codes involve a level of physician involvement. Although a code exists for a *stand-alone screening tool*, no such code exists to cover the costs associated with a *stand-alone treatment tool*, such as app-based cognitive behavioral therapy (CBT). As such, a CBT tool would need to be reimbursed (1) on the basis of the brief screenings that it may contain to assess patient progress, (2) on the basis of the human services that are wrapped around it, or (3) through a noncode payment channel. The first case is problematic, as it undervalues the curative effects of the app, beyond mere screening. The second case is problematic, as it requires human intervention and limits the ability of mental health to scale via technology. Similarly, the third case is problematic, as it requires the app developer to negotiate a direct payment or obtain US Food and Drug Administration (FDA) approval for classification as a prescription device or drug. When apps are used in conjunction with physician or technician effort to achieve the requirements of a CPT code, there are typically time requirements that are used to determine the extent to which a CPT code is paid. More is paid when greater quantities of physician or technician time are required. As such, *these time requirements hamper the introduction of technology-based efficiency into mental health*, as if certain time thresholds are not met, some codes become unbillable. A relaxation of these time requirements or the introduction of more codes requiring 0 min of clinician or technician time would create new avenues for the introduction of efficiency through technology. Given the shortage of mental health providers, yoking payment to the direct involvement of humans (physicians or nonphysicians) is a barrier to efficiency and increased provider capacity.

### Apps as Devices and Drugs

To overcome the linkage between CPT-based reimbursement and physician work, malpractice, and practice expenses, some organizations have chosen to pursue other paths of reimbursement. Namely, as the existing mechanisms for reimbursing devices and drugs do not consider the degree of physician effort when determining reimbursements, these mechanisms are being pursued by several app companies. When apps are treated as prescription devices or drugs, they may only be billed by individuals with prescribing authority. States vary in their willingness to grant various nonphysician health care providers, from nurse practitioners to psychologists, the ability to prescribe, and in some cases, grant only partial prescribing authority. As a result of the limitations on prescribing authority, a majority of psychologists are unable to prescribe apps, as most of them lack prescribing authority [7]. Nonetheless, organizations pursuing this route may see the rigor of FDA approval as a stamp of quality and a potential barrier to entry for competitors. Furthermore, as FDA approval is generally required for apps acting as medical devices or as accessories to them, in some cases, FDA approval is pursued out of necessity [8]. There are a number of organizations pursuing the device and drug approach. Working with Sandoz, a pharmaceutical company, Pear Therapeutics obtained FDA clearance for its app for patients with substance use disorder as the first prescription digital therapeutic [9]. Meanwhile, Click Therapeutics partnered with Otsuka to bring to market an app for major depressive disorder, which it intends to have classified as software as a medical device for the purposes of regulation by the FDA [10]. Akili Interactive Labs has sought FDA approval for its attention-deficit/hyperactivity disorder intervention, which it deems a “digital medicine,” and has worked with the pharmaceutical manufacturer Shionogi to commercialize it in Asia [11]. In all 3 cases, partnerships with external pharmaceutical companies were forged.

### Gaps in Existing Reimbursement Pathways Limiting the Reimbursement of Some Apps

Although there are multiple potential ways in which apps can be fit into the existing reimbursement system, there is no guarantee of payment for most codes; time from a health care provider or a technician may be necessary to achieve reimbursement, and the app, in some situations, may need to undergo a regulatory review process for it to be reimbursed if used by patients on a stand-alone basis. When apps can only be reimbursed when these various accommodations are made, it adds friction to their development and utilization. Furthermore, time-based requirements for human participation limit the degree to which technology can drive efficiency in mental health.

### Limited Support for Self-Directed Treatment

As was mentioned during the discussion of how RVUs are assigned to CPT codes, when an app does not involve clinician or technician activity, it is not likely to receive substantial reimbursement. Thus, some developers have sought to have their apps covered as drugs or devices. HCSPCS codes, such as T1505, electronic medication compliance management device, include all components and accessories, not otherwise classified; A9999, miscellaneous durable medical equipment supply or accessory, not otherwise specified; and E1399, durable medical equipment, miscellaneous, provide the sorts of catchall pathways necessary for apps seeking a durable medical equipment approach to reimbursement. There may be 2 pathways toward creating simpler reimbursement mechanisms for app-related treatments. First, the existing durable medical equipment codes could be clarified to include apps. Although they are already being used in some cases for this purpose, they are at the discretion of the insurer. Second, there may be a need for a treatment-related equivalent to the brief emotional/behavioral assessment code 96127. Such a code could be used to cover automated app-based treatment conducted in a standardized fashion rather than automated app-based screening.

### Limitations on Administration of Measures and Patient Cost Exposure

Screenings for conditions, such as depression (eg, Patient Health Questionnaire-9) and anxiety (eg, General Anxiety Disorder-7), are the laboratory tests of mental health. Screenings can be performed repeatedly on the same patients to facilitate measurement-based care. There is substantial evidence to show that the frequent use of such screenings to implement measurement-based care is beneficial to patient welfare, as they enable mental health care providers to better understand the trajectory of an illness [12]. Although the CPT code 96127 does provide a potential means of covering the cost of measurement-based care, there are sometimes limitations on its frequency of use and on the number of screening exams that may billed during a given visit. These limitations vary by payer. For instance, Amerigroup allows a maximum of 2 units of 96127 per visit [13]. PerformCare allows 96127 to be billed up to 4 units per day, every day [14]. In 2018, Aetna removed a restriction that had been placed on the code, which had only allowed it to be billed once per year [15]. These limitations are in direct conflict with multiple research studies that document that these standardized measures need to be administered frequently to properly guide treatment decisions and improve outcomes [12]. The lack of uniformity in reimbursement policies for this code across payers may impact app developers, as patients with some health plans may yield substantially more revenue as a result of this code than patients with other health plans.

Although there are benefits to screening for patients, the use of app-based screening has the potential to result in frequent and, perhaps, unanticipated copayments. Although the code 96127 was originally implemented and created to support the Affordable Care Act’s mandate to include mental health services as a component of required Essential Health Benefits, the use of the code only qualifies for the Affordable Care Act’s no-cost sharing provision if it is billed as a screening for an asymptomatic patient (patient with the International Classification of Diseases, version 10 code Z13.89) [16]. Patients who are symptomatic and are using screening tools as a component of ongoing measurement-based care are potentially subject to copayments. To avoid surprising patients with bills related to screenings that they self-administered, it is necessary for health care providers to carefully explain the financial consequences of the use of this code or consider avoiding billing it. As self-administered, reimbursable tools for mental health become more prevalent, there will inevitably need to be substantial patient education regarding the costs associated with the tools. If repeated screening leads to substantially improved outcomes and cost savings, payers may wish to eliminate copayment requirements for screening apps to improve adherence.

### Conclusions

There is no standard pathway through which mental health apps can all be reimbursed. The appropriate pathway is dependent on the nature of the app and its degree of clinician and technician involvement. The costs associated with a number of apps are actively being reimbursed today, both directly and indirectly. Nonetheless, substantial friction to reimbursement remains, and many apps are funded through out-of-pocket payments by patients. As apps are being used to support various activities within mental health care, including therapy sessions, care management, case management, and collaborative care, in many cases, reimbursement is occurring for a bundle of services, which is app facilitated, rather than for the app itself. Going forward, there are a number of changes to the reimbursement system, which could facilitate the adoption of digital tools for mental health. Namely, the FDA’s approval process for apps could be simplified and standardized, and CPT codes could be created or modified to facilitate payments for additional services where there are 0 min of clinician or technician time involved in service delivery.

## Abbreviations

CBT: cognitive behavioral therapy
CoCM: collaborative care model
CPT: Current Procedural Terminology
FDA: US Food and Drug Administration
HCSPCS: Healthcare Common Procedure Coding System
RVU: Relative Value Unit

## References

[1] Fortney JC, Unützer J, Wrenn G, Pyne JM, Smith GR, Schoenbaum M, Harbin HT (2017). A tipping point for measurement-based care. Psychiatr Serv.

[2] AAP Division of Health Care Finance (2016). AAP Gateway.

[3] CorrectCodeChek - DecisionHealth.

[4] DeAnn D (2018). Avicenna Medical Systems.

[5] American Medical Association (2018). CPT Professional 2019.

[6] Beck DE, Margolin DA (2007). Physician coding and reimbursement. Ochsner J.

[7] American Psychological Association Services.

[8] (2015). US Food and Drug Administration.

[9] (2018). Pear Therapeutics.

[10] (2019). Business Wire.

[11] (2019). Akili Interactive.

[12] Fortney J, Sladek R, Unutzer J, Kennedy P, Harbin H, Emmet B, Alfred L, Carneal G (2015). The Kennedy Forum.

[13] (2018). Providers – Amerigroup.

[14] Daubert S (2016). PING PDF Document Flatform.

[15] Kressly SJ (2019). AAP Gateway.

[16] (2018). Connected Mind.

